# The Temporal Relationship Between Faulty Gambling Cognitions and Gambling Severity in Young Adults

**DOI:** 10.1007/s10899-016-9605-y

**Published:** 2016-04-13

**Authors:** Ryan Nicholson, Chad Graves, Michael Ellery, Tracie O. Afifi

**Affiliations:** 1 Department of Psychology, University of Manitoba, Winnipeg, Canada; 2 Departments of Community Health Sciences and Psychiatry, University of Manitoba, S113-750 Bannatyne Avenue, Winnipeg, MB R3E 0W5 Canada

**Keywords:** Pathological gambling, Young adult gambling, Gambling cognitions, Longitudinal gambling research, Gambling development, Gambling severity

## Abstract

Disordered gambling in young adults is hypothesized as being related to mistaken gambling-related cognitions. Few studies have examined the temporal order of this relationship using longitudinal data. The purpose of this study is to understand the directionality of the relationship between gambling cognitions and gambling severity in a longitudinal sample of young adults. Young adults (N = 578), initially aged 18–21 years, completed the Manitoba Longitudinal Survey of Young Adults at two time points approximately 2–3 years apart. Measures of beliefs about randomness related to gambling and gambling severity, as measured by the Problem Gambling Severity Index, were utilized. A cross-sectional relationship between gambling severity and gambling-related cognitions was observed with greater gambling severity being associated with increased endorsement of mistaken cognitions. Evidence for a bidirectional longitudinal relationship was observed with faulty gambling cognitions leading to later problematic gambling behaviors and vice versa when examining a total beliefs scale. When examining specific beliefs about randomness, initial gambling group membership predicted later endorsement of certain beliefs about randomness while initial belief ratings did not impact later gambling group membership. The results of this study suggest a bidirectional relationship between gambling severity and erroneous gambling-related cognitions. However, when examining specific beliefs about randomness, evidence was found for problem gambling behaviors leading to erroneous gambling beliefs. These findings suggest that prevention efforts targeting cognitions may not be as effective in impacting those not yet demonstrating disordered gambling behaviors.

## Introduction

Studies of pathological and at-risk gambling have identified gambling at younger ages as a risk factor in the development of a gambling problem (Derevensky et al. 2003). Research on gambling in young adults (19–25 year olds) has found 67–97 % of this age group participated in some form of gambling (Clarke 2003; Engwall et al. 2004) with prevalence rates estimating that 5 % of young adults are pathological gamblers (Shaffer et al. 1999), a rate of three times higher than the general population (Shaffer and Hall 2000; Shaffer et al. 1997; Volberg 1996). This is particularly problematic as problem gambling during young adulthood is associated with a number of negative consequences, including poor academic performance, depression, suicide, and the development of multiple addictions (Afifi et al. 2016; Engwall et al. 2004; Ladouceur et al. 1994; Lesieur et al. 1991; Stuhldreher et al. 2007).

The high prevalence of problem gambling found in young adults and its negative implications has led to greater efforts to identify the factors underlying it, with faulty gambling cognitions being identified as a risk factor in the development of problem gambling behaviors (Clarke 2003; Engwall et al. 2004; Stuhldreher et al. 2007). Faulty gambling cognitions focus on the gambler’s own beliefs regarding their control or influence over gambling outcomes (Joukhador et al. 2003) with many gamblers believing they have some degree of control when, in fact, most forms of gambling capitalize on randomness. Other forms of these distorted cognitions include denial, superstition, and overconfidence in perceived skill. While problem gamblers mistakenly attribute their winnings to internal factors such as superstitious behaviour and perceived skill, losses are explained away as result of external factors (Ladouceur et al. 1988; Langer 1975; Wagenaar 1988).

Extending from examinations of behaviors, recent research has demonstrated a positive relationship between gambling severity and gambling cognitions (Lakey et al. 2007; MacKay and Hodgins 2012; Oei et al. 2008). Pathological gamblers frequently endorse erroneous gambling beliefs leading to overconfidence in their bets and have demonstrated a short-term focus on rewards with insensitivity to future consequences (Lakey et al. 2007). Further, these gambling cognitions significantly predicted pathology according to DSM-IV criteria (Lakey et al. 2007). This association between faulty gambling cognitions and gambling severity extends to pathological or at-risk gambling behaviors in young adults (e.g., Hardoon and Derevensky 2002; Moodie 2008; Moore and Ohtsuka 1999). Research on young adult gamblers has shown a significant association between problem gambling severity and faulty cognitions with problem gamblers endorsing higher rates in their belief of luck, more positive attitudes towards gambling, and more erroneous beliefs on luck and perseverance than non-problem gamblers (Chiu and Storm 2010; MacKillop et al. 2006). While these studies demonstrate a significant association between faulty gambling cognitions and gambling severity in young adults, the research conducted to this point has largely been limited to using cross-sectional data (e.g., Ladouceur 2004; MacKay and Hodgins 2012; May et al. 2005), with few longitudinal studies conducted (Grant et al. 2012; Harvanko et al. 2013).

Complicating the relationship between faulty cognitions and gambling severity, some studies have found that irrational gambling cognitions have no effect on gambling behaviors while gambling (Cronce and Corbin 2010; Ellery and Stewart 2014; May et al. 2005). Specifically, changes to gambling cognitions of gamblers did not affect the number of bets made during the gambling session, total amount of money won or lost during the session, total amount of money wagered during the session, average amount of money won or lost per bet, or average amount of money wagered per bet (May et al. 2005). This finding suggests that factors apart from faulty cognitions must be considered in the development of problem gambling behaviors. However, change in illusion of control was relatively small, which may explain the lack of change in gambling behavior. Further, and perhaps more importantly, the sample utilized largely non-problem gamblers who did not exhibit risky gambling behaviors or patterns.

Further inconsistencies in the research literature cast doubt on whether irrational cognitions about gambling leading to increased gambling severity, with some suggesting that increased problem gambling behaviors precede the development of irrational beliefs about gambling (Ellery and Stewart 2014). In support of this view, it has been shown that winning during a gambling session increases the endorsement of irrational beliefs about gambling (Monaghan et al. 2010).

A potential reasoning for the discrepant findings within the belief versus severity research may be related to within play tendencies. Many of the articles previously described (e.g., Ellery and Stewart 2014; May et al. 2005; Monaghan et al. 2010) examined the changes in beliefs during a single session of gambling rather than the long-term development of severity or belief changes over a longer span. These discrepant findings suggest that further research is needed to better understand the directionality of the relationship between gambling cognitions and problem gambling severity.

## Present Study

The current study aims to clarify inconsistencies within the gambling literature about the directionality of the relationship between gambling cognitions and gambling severity by analyzing the development of gambling beliefs, attitudes, and fallacies over time and comparing this development to the development of gambling problems over time. This study examined the gambling severity and gambling related cognitions of young adults from Manitoba at two time periods approximately two-to-3 years apart.

## Research Questions

1. Are gambling problems associated with faulty gambling cognitions cross-sectionally?
2. Do gambling problems predict later faulty gambling cognitions, after adjusting for initial cognitions?
3. Do faulty gambling cognitions predict later gambling problems, after adjusting for initial gambling problems?

## Methods

### Participants

The current study utilized data from the Manitoba Longitudinal Study of Young Adults (MLSYA). The MLSYA dataset was created through the collaboration between the Manitoba Gaming Control Commission, the Addictions Foundation of Manitoba, and the Manitoba Lotteries Corporation. Young adults from Manitoba, aged 18–20 at baseline, were surveyed at four time points across a 5-year span from 2007 to 2011. Initially, 679 young adults complete the survey at the first time point. Of the 679 participants to complete the study at baseline, 578 (85.1 %) went on to complete the survey at both baseline and follow-up and, therefore, met inclusion criteria.

At baseline, participants were 18 (35.6 %), 19 (36.8 %), or 20 (27.5 %) years old with a mean age of 18.9 years. At follow-up, 24.9 % of participants were 20 years old, 36.9 % were 21, 31.1 % were 22, and 7.1 % were 23 years old or more. The mean age at follow-up was 21.2 years. At baseline, 51.8 % of the sample was female. The mean age of males and female at baseline were 18.96 years (SD = 0.78) and 18.88 years (SD = 0.80) respectively. Sociodemographic characteristic differences by gambling severity at baseline were only found for sex and main activity in the past 12 months, with males being more likely to be a moderate or severe risk gamblers and those working or indicating other as their main activity as being more likely to be a moderate or severe risk gambler (see Table 2).

### Procedure

Respondents were recruited through convenience, random, and snowball sampling. Specific sampling procedures utilized by the MLSYA included random-digit dialing, internet advertising, and advertisements in casinos, VLT locations, and post-secondary institutions. In addition, participants were asked for potential referrals post-interview. The characteristics of the young adults at baseline of the MLSYA was similar to the sociodemographic characteristics of Manitobans aged 18–20 years, with the exception of MLSYA participants noting slightly higher education and residing predominantly in urban areas. A two-part survey was administered to respondents during Cycle 1, with the first part consisting of a telephone interview and the second part involving the respondents’ choice of an online or mail-in questionnaire. Telephone interviews were utilized in the follow-up surveys (i.e., Cycles 2–4).

The MLSYA includes data from four time points, or cycles, over a 5-year period from 2007 to 2011. Measures related to gambling were assessed at Cycle 1 (in 2007) and Cycle 3 (2009–2010) only. For the purposes of this study, Cycle 1 and 3 are referred to as baseline and follow-up respectively and represent an approximate 2–3 year interval between these time points.

### Measures

#### Demographics

The MLSYA questionnaire collected participants’ sociodemographic information including age, gender, marital status, main activity engaged in over the past year (e.g., work or school), religion, self-identified ethnicity, and total household income in the past 12 months.

#### Gambling Problems

This research utilized the Problem Gambling Severity Index (PGSI), a subscale of the Canadian Problem Gambling Index (CPGI), to assess past 12-month prevalence of problem gambling. The PGSI uses nine items to assess the severity of gambling problems: (1) wagered larger amounts to get the same feeling of excitement, (2) tried to win back losses, (3) borrowed money or sold something to get money for gambling, (4) felt you might have a problem with gambling, (5) gambling caused health problems including stress and anxiety, (6) been criticized for your betting or told that you have a problem, (7) gambling has caused financial problems, (8) felt guilty about gambling, and (9) bet more than you could afford to lose. The respondent indicates how frequent each of the above behaviors or problems occurred during the past 12 months: never, sometimes, most of the time, or almost always.

Previous psychometric testing of the PGSI established specific categories of gamblers based on their scores. Those who did not gamble at least five times in the past year were noted as being non-gamblers. Of those who did gamble the requisite frequency in the past 12 months, four categories were created, consisting of non-problem gamblers (score of zero), low risk gamblers (score of one to two), moderate risk gamblers (score of three to seven), and severe risk gamblers (score of eight or more) (Ferris and Wynne 2001a, b). While the developers of the PGSI indicate low- and moderate-risk as distinct groups with unique cut-off points and associated characteristics, other research on the PGSI suggests the two groups lack meaningful contrast and should be merged into one group (Currie et al. 2013). For the purposes of this study, we utilized the cut-off points more reflective of the original Ferris and Wynne designations. Therefore, this study utilized two gambling categories: ‘non-gamblers and low-risk gamblers’ (i.e., non-gamblers, non-problem gamblers scoring zero on the PGSI, and low-risk gamblers scoring 1 or 2 on the PGSI) and participants indicating three or more problem gambling symptoms according to the PGSI (i.e., moderate and severe risk gamblers) termed ‘moderate to severe risk gamblers’.

### Gambling Beliefs and Attitudes

To examine gambling beliefs, attitudes, and fallacies, data collected using the Manitoba Gaming Control Commission’s (MGCC) gambling attitudes and fallacies questionnaire (MGCC 2007) and the Drake Beliefs About Chance Inventory (Wood and Clapham 2005) was employed. The MGCC questionnaire was created by the Gaming Commission as a means of assessing whether participants ‘agree’ or ‘disagree’ with erroneous gambling statements about randomness. These items include ‘Odds of winning on a slot machine change as you are playing’, ‘A series of numbers like 12–5–23–7 is more likely to win than 1–2–3–4’, and ‘Staying at the same slot machine will improve your chances of winning’. The items on the MGCC questionnaire and individual item mean and standard deviation scores are listed in Table 1.

**Table 1:** Beliefs about randomness surveyed in the MLSYA

	Gambling related cognition	Baseline mean (SD)	Follow-up mean (SD)
Beliefs about randomness			
BAR 1	The odds of winning on a slot machine change as you are playing	0.25 (0.44)	0.13 (0.34)
BAR 2	It is important to understand exactly how a slot machine or VLT works in order to play better	0.39 (0.49)	0.23 (0.42)
BAR 3	Having a system when playing slot machines or VLTs increases the chances of winning	0.11 (0.31)	0.10 (0.31)
BAR 4	Staying at the same slot machine or VLT will improve your chances of winning	0.09 (0.29)	0.06 (0.24)
BAR 5	If you have been losing for a while, odds are you are due for a win	0.11 (0.31)	0.05 (0.22)
BAR 6	If you flip a coin and get heads five times in a row, your next flip is likely to be tails	0.21 (0.41)	0.10 (0.31)
BAR 7	A series of numbers such as 12–5–23–7 is more likely to win than a series of numbers like 1–2–3–4	0.23 (0.42)	0.11 (0.32)
Drake Total Beliefs Scale		41.18 (13.94)	37.28 (13.64)
Drake Superstition Subscale	e.g., ‘I can improve my chances of winning by performing specific rituals’. or ‘I believe that fate is against me when I lose’	20.07 (7.68)	18.44 (7.49)
Drake Illusion of Control Subscale	e.g., ‘There are secrets to successful casino gambling that can be learned’. or ‘One should pay attention to lottery numbers that often win’	21.11 (8.11)	18.84 (7.85)

The Drake Beliefs About Chance Inventory (Wood and Clapham 2005) is a 22-item survey designed to assess two commonly observed areas of faulty gambling cognitions: superstition and illusion of control. Responses to items were given on a 5-point Likert scale from 1 (strongly disagree) to 5 (strongly agree). Examples of items included to assess superstition include ‘I can improve my chances of winning by performing specific rituals’, and ‘I believe that fate is against me when I lose’. Examples of items from the illusion of control subscale include ‘There are secrets to successful casino gambling that can be learned’, or ‘One should pay attention to lottery numbers that often win’. Of the 22-items, half made up the superstition subscale while the other half comprised the illusion of control subscale. Individual items from the MGCC questionnaire and the superstition subscale score, the illusion of control subscale score, and the total score of the Drake inventory were examined in the analyses. Subscale score means and standard deviations for baseline and follow-up are located in Table 1.

### Statistical Analyses

Descriptive statistics at baseline were examined for sociodemographic variables among ‘non-gamblers and low-risk gamblers’ and ‘moderate- to severe-risk gamblers’. Cross-sectional logistic regressions were utilized to examine the strength of the relationship between baseline cognition ratings/responses and baseline gambling group membership. Models were run without adjusting for any other variables (unadjusted Odds Ratios, OR) and again with adjustment for sociodemographic variables (Adjusted Odds Ratios, AOR).

Logistic regressions analyses were conducted to assess for a temporal relationship between gambling group and faulty gambling cognitions. Unadjusted models, models adjusting for sociodemographic variables (AOR-1), and models adjusting for both sociodemographic variables and initial group membership or cognition responses (AOR-2) were conducted.

## Results

At baseline (N = 679), 11.5 % of the sample reported not participating in the prerequisite number of gambling activities in the past year, 57.0 % were non-problem gamblers (PGSI score of 0), 20.8 % were low-risk gamblers (score of 1–2), 9.3 % were moderate-risk gamblers (score of 3–7), and 1.5 % were problem gamblers (score of 8 or more). When examining the collapsed gambling groups, 89.2 % of the respondents were non-gamblers or low-risk gamblers (i.e., score of 2 or less) while 10.8 % were moderate to severe risk gamblers (i.e., score of 3 or more). At follow-up (N = 578), 8.5 % of respondents did not gamble at least five times in the past year while 71.8 % were non-problem gamblers, 13.5 % were low-risk gamblers, 4.7 % were moderate-risk gamblers, and the remaining 1.6 % fell into the severe-risk gambler category.

The sociodemographic variables of the gambling groups are presented in Table 2. Differences between the two gambling groups were observed in sex and main activity engaged in over the past year. Males were more likely to be a moderate to severe risk gambler (64.4 %) compared to females (35.6 %). Moderate to severe risk gamblers, compared to non-gamblers and low-risk gamblers, were more likely to report work (35.6 vs. 25.4 %) as their main activity and less likely to their report main activity as school (57.5 vs. 71.3 %).

**Table 2:** Sociodemographic variables of gambling group membership at baseline

	Non-gambler or non-problem gambler (N = 606; 89.2 %)	At-risk or problem gamblers (N = 73; 10.8 %)
Marital status	χ^2^ = 0.489, *p* = .783	
Single (never married)	400 (66.0 %)	51 (69.9 %)
In a relationship	194 (32.0 %)	21 (28.8 %)
Married/common-law	12 (2.0 %)	1 (1.4 %)
Divorced/separated/widowed	–	–
Sex	χ^2^ = 8.624, *p* = .003	
Female	326 (53.8 %)	26 (35.6 %)
Male	280 (46.2 %)	47 (64.4 %)
Main activity past 12 months	χ^2^ = 8.290, *p* = .040	
School	432 (71.3 %)	42 (57.5 %)
Working	154 (25.4 %)	26 (35.6 %)
Looking for work	9 (1.5 %)	1 (1.4 %)
Other	11 (1.8 %)	4 (5.5 %)
Total household income before taxes—past 12 months	χ^2^ = 5.315, *p* = .806	
<$10,000	9 (1.5 %)	3 (4.1 %)
$10,001–$19,999	5 (0.8 %)	1 (1.4 %)
$20,000–$29,999	18 (3.0 %)	1 (1.4 %)
$30,000–$39,999	14 (2.3 %)	1 (1.4 %)
$40,000–$49,999	15 (2.5 %)	1 (1.4 %)
$50,000–$59,999	20 (3.3 %)	2 (2.7 %)
$60,000–$79,999	44 (7.3 %)	3 (4.1 %)
$80,000–$99,999	39 (6.4 %)	6 (8.2 %)
$100,000+	177 (29.2 %)	22 (30.1 %)
DK/NR	265 (43.7 %)	33 (45.2 %)
First identified ethnic group (other than Canadian)	χ^2^ = 1.671, *p* = .643	
European	417 (68.8 %)	47 (64.4 %)
Asian	50 (8.3 %)	9 (12.3 %)
Other	90 (14.9 %)	10 (13.7 %)
DK/NR	49 (8.1 %)	7 (9.6 %)
Religion	χ^2^ = 1.859, *p* = .762	
No religion/agnostic/atheist	235 (38.8 %)	29 (39.7 %)
Christian	106 (17.5 %)	10 (13.7 %)
Roman Catholic	87 (14.4 %)	12 (16.4 %)
All others	165 (27.2 %)	19 (26.0 %)
DK/NR	13 (2.1 %)	3 (4.1 %)

The results of the cross-sectional regression analyses are displayed in Table 3. The results indicate that moderate to severe risk gamblers, when compared to non-gamblers and low-risk gamblers, are more likely to report certain faulty gambling cognitions. Those in the moderate to severe risk gambler group were more likely than the non-gamblers and low-risk gambling group to endorse a faulty gambling cognition about randomness in four of the seven beliefs about randomness assessed after adjusting for sociodemographic factors. Similarly, scores on the overall Drake Total Beliefs scale and both the Superstition and Illusion of Control subscales were reflective of gambling-related cognitive errors in moderate to severe risk gamblers compared to non-gamblers and low-risk gamblers.

**Table 3:** Cross-sectional analysis examining relationship between baseline gambling group membership and baseline gambling cognition endorsement

Gambling related cognitions	At-risk or problem gamblers	
	OR (95 % CI)	AOR (95 % CI)
BAR 1: The odds of winning on a slot machine change as you are playing	1.21 (0.71–2.08)	1.23 (0.72–2.23)
BAR 2: It is important to understand exactly how a slot machine or VLT works in order to play better	1.31 (0.80–2.14)	1.36 (0.82–2.26)
BAR 3: Having a system when playing slot machines or VLTs increases the chances of winning	3.06 (1.66–5.64)***	2.97 (1.55–5.68)**
BAR 4: Staying at the same slot machine or VLT will improve your chances of winning	2.44 (1.25–4.75)**	2.60 (1.28–5.28)**
BAR 5: If you have been losing for a while, odds are you are due for a win	2.20 (1.16–4.17)*	2.38 (1.22–4.66)*
BAR 6: If you flip a coin and get heads 5 times in a row, your next flip is likely to be tails	2.03 (1.20–3.44)**	2.12 (1.22–3.67)**
BAR 7: A series of numbers such as 12–5–23–7 is more likely to win than a series of numbers like 1–2–3–4	1.43 (0.83–2.47)	1.36 (0.77–2.41)
Drake Total Beliefs Scale	1.04 (1.02–1.06)***	1.04 (1.02–1.05)***
Drake Superstition Subscale	1.06 (1.02–1.09)***	1.06 (1.03–1.09)***
Drake Illusion of Control Subscale	1.06 (1.03–1.10)***	1.06 (1.03–1.09)***

‘Non-gambler or non-problem gambler’ serves at the reference group. *AOR* adjusting for sociodemographic variables (i.e., gender, marital status, main past-year activity, religion, ethnicity, total household income in past-year)

* *p* < 0.05; ** *p* < 0.01; *** *p* < 0.001

Table 4 displays the regression results examining the association between gambling group membership at baseline and reported faulty gambling cognitions at follow-up. After adjusting for sociodemographic factors, the endorsement of four out of seven beliefs about randomness at follow-up differed between the two baseline gambling groups. The endorsement of BAR5, ‘If you have been losing for a while, odds are you are due for a win’, (AOR2 = 3.18; 95 % CI 1.23–8.96) significantly differed between the two baseline gambling groups after adjusting for both sociodemographic factors and respective reported BAR scores at baseline. The Drake Total Beliefs scale and both the Superstition and the Illusion of Control subscales were significantly different at follow-up between the two baseline gambling groups at follow-up after being adjusted for sociodemographic factors. After adjusting for baseline belief endorsement, only the follow-up Drake Superstition subscale scores demonstrated a difference between baseline gambling groups [adjusted unstandardized B coefficient-2 (AB-2) = 1.87; 95 % CI 0.16–3.57].

**Table 4:** Longitudinal analysis examining relationship between baseline gambling group membership and follow-up gambling cognition endorsement

Gambling related cognitions	At-risk or problem gamblers		
	OR (95 % CI)	AOR (95 % CI)	AOR-2 (95 % CI)
BAR 1: The odds of winning on a slot machine change as you are playing	2.09 (1.07–4.09)*	2.07 (1.00–4.25)*	2.11 (0.98–4.55)
BAR 2: It is important to understand exactly how a slot machine or VLT works in order to play better	1.17 (0.63–2.19)	1.23 (0.64–2.36)	1.21 (0.59–2.48)
BAR 3: Having a system when playing slot machines or VLTs increases the chances of winning	2.23 (0.93–5.34)	2.07 (0.79–5.46)	1.79 (0.65–4.97)
BAR 4: Staying at the same slot machine or VLT will improve your chances of winning	2.31 (0.97–5.55)	3.18 (1.22–8.28)*	2.65 (0.97–7.26)
BAR 5: If you have been losing for a while, odds are you are due for a win	3.80 (1.60–9.03)**	3.99 (1.55–10.24)**	3.18 (1.23–8.96)*
BAR 6: If you flip a coin and get heads 5 times in a row, your next flip is likely to be tails	2.24 (1.09–4.60)*	2.47 (1.14–5.36)*	1.98 (0.87–4.51)
BAR 7: A series of numbers such as 12–5–23–7 is more likely to win than a series of numbers like 1–2–3–4	1.08 (0.47–2.48)	1.33 (0.56–3.20)	1.22 (0.48–3.08)

	B (95 % CI)	Adjusted B (95 % CI)	Adjusted B-2 (95 % CI)
Drake Total Beliefs Scale	6.55 (2.84–10.25)**	6.09 (2.34–9.84)**	2.12 (−0.94 to 5.18)
Drake Superstition Subscale	3.71 (1.68–5.75)***	3.68 (1.63–5.74)***	1.87 (0.16 to 3.57)*
Drake Illusion of Control Subscale	2.83 (0.69–4.98)*	2.41 (0.25–4.56)*	0.51 (−1.31 to 2.34)

‘Non-gambler or non-problem gambler’ serves at the reference group. *AOR* adjusting for sociodemographic variables (i.e., gender, marital status, main past-year activity, religion, ethnicity, total household income in past-year). *AOR-2* adjusting for sociodemographic variables and baseline gambling cognition endorsement

* *p* < 0.05; ** *p* < 0.01; *** *p* < 0.001

The logistic regression results examining baseline gambling cognitions leading to follow-up gambling group membership is shown in Table 5. None of the beliefs about randomness items at baseline predicted later gambling group membership at follow-up in any of the models examined. The Drake Total Beliefs scale and the Superstition and Illusion of Control subscales all predicted later gambling group membership after adjusting for sociodemographic characteristics in the first adjusted models (AOR); however, these relationships did not remain significant after adjusting for gambling group membership at baseline in the second adjusted models (AOR-2).

**Table 5:** Longitudinal analysis examining relationship between baseline gambling cognition endorsement and follow-up gambling group membership

Gambling related cognitions	At-risk or problem gamblers		
	OR (95 % CI)	AOR (95 % CI)	AOR-2 (95 % CI)
BAR 1: The odds of winning on a slot machine change as you are playing	0.83 (0.37–1.86)	0.91 (0.38–2.15)	0.78 (0.31–1.96)
BAR 2: It is important to understand exactly how a slot machine or VLT works in order to play better	0.87 (0.43–1.74)	0.90 (0.43–1.91)	0.81 (0.36–1.83)
BAR 3: Having a system when playing slot machines or VLTs increases the chances of winning	2.16 (0.91–5.12)	2.45 (0.93–6.47)	1.54 (0.53–4.48)
BAR 4: Staying at the same slot machine or VLT will improve your chances of winning	1.29 (0.44–3.78)	1.84 (0.55–6.24)	1.28 (0.35–4.69)
BAR 5: If you have been losing for a while, odds are you are due for a win	1.36 (0.51–3.62)	2.04 (0.70–5.92)	1.22 (0.36–4.12)
BAR 6: If you flip a coin and get heads 5 times in a row, your next flip is likely to be tails	1.09 (0.48–2.44)	1.53 (0.64–3.64)	1.17 (0.46–2.98)
BAR 7: A series of numbers such as 12–5–23–7 is more likely to win than a series of numbers like 1–2–3–4	1.01 (0.45–2.27)	0.94 (0.39–2.22)	0.82 (0.32–2.13)
Drake Total Beliefs Scale	1.04 (1.01–1.06)**	1.03 (1.01–1.06)**	1.02 (1.00–1.05)
Drake Superstition Subscale	1.04 (1.00–1.09)*	1.05 (1.00–1.09)*	1.03 (0.99–1.08)
Drake Illusion of Control Subscale	1.06 (1.03–1.11)**	1.06 (1.01–1.10)*	1.04 (1.00–1.09)

‘Non-gambler or non-problem gambler’ serves at the reference group. *AOR* adjusting for sociodemographic variables (i.e., gender, marital status, main past-year activity, religion, ethnicity, total household income in past-year). *AOR-2* adjusting for sociodemographic variables and baseline gambling group membership

* *p* < 0.05; ** *p* < 0.01; *** *p* < 0.001

## Discussion

Consistent with previous research, gambling severity was found to be associated with faulty gambling cognitions in a cross-sectional analysis. Longitudinal analyses provide evidence for a directional relationship between initial gambling severity group membership and specific later faulty gambling cognition endorsements, In contrast, the ratings for the beliefs about randomness variables showed no relationship between initial faulty gambling cognition endorsement and later gambling severity group membership. These results suggest that, over time, those with moderate to severe risk gambling behaviors may be associated with an increased likelihood of erroneous concepts around gambling and associated odds.

The research literature has extensively demonstrated a positive relationship between faulty gambling cognitions and increased gambling severity (Lakey et al. 2007; MacKay and Hodgins 2012; Oei et al. 2008). Overall, problem gamblers have consistently shown to hold more faulty cognitions related to luck, superstition, and illusion of control than non-problem gamblers (Oei et al. 2008). While this relationship has been well established, evidence for the directionality of the relationship to this point has been largely unknown. Our findings when examining specific beliefs about randomness suggest that early gambling problems are associated with later faulty gambling cognitions, not the opposing relationship. However, more broad assessments of mistaken cognitions, including superstition and illusion of control, suggest that a bidirectional relationship may cause faulty beliefs to further gambling severity while, simultaneously, increased severity furthers the development of faulty gambling cognitions.

Previous theories may shed light on this seemingly dual finding. Sharpe (2002) has theorized a potential pathway to how gambling involvement leads to the development of faulty gambling cognitions. She proposes that early history (e.g., big wins) and exposure to gambling leads to the development of these cognitions and the establishment of gambling behavior. Through classical conditioning, the faulty cognitions are paired with the arousal and the cues associated with gambling. Overtime, this conditioned response becomes automatic (McCusker and Gettings 1997). Erroneous gambling beliefs are then unknowingly triggered by cues associated with gambling and, as a result of this learning process, leads to more frequent and prolonged gambling sessions.

Another theory posits that disordered gambling is a result of erroneous decision-making based on faulty information processing (Ladouceur and Walker 1996; Sharpe 2002; Sharpe and Terrier 1993). This cognitive model suggests that problem gamblers have faulty cognitive heuristics or biases, which leads to the development of problem gambling behaviors. Similarly, the results of the current study suggest that cognitions may play a role in both the initiation of problematic gambling behavior and maintenance of problem gambling behaviors. Previously, gambling cognitions have been found to moderate the relationship between risky gambling practices and gambling intensity (Miller and Currie 2008). Specifically, these cognitions were found to increase the percentage of income spent on gambling by individuals who engage in risky behaviors and were associated with gamblers needing a higher dose (e.g., frequency, intensity) of gambling to be satiated. Likewise, Delfabbro and Winefield (2000) described a moderating effect of gambling cognitions whereby non-problem gamblers, with higher rates of faulty gambling cognitions, were more likely to spend a larger amount of money in a gambling session.

## Implications

An important implication of this study’s findings pertains to the prevention and treatment of disordered gambling. Many intervention programs aim to identify and challenge related cognitions through clinical intervention (Ferland et al. 2002; Ladouceur et al. 2000, 2001, 2003). The central assumption underlying these cognitive interventions assumes that the correction of erroneous beliefs will reduce problem gambling. This is supported in the research literature, where efforts to change cognitions have been more successful in reducing problematic gambling behaviors rather than preventing future gambling disorders (Benhsain et al. 2004; Ferland et al. 2002; Ladouceur et al. 2000, 2001, 2003; Williams et al. 2007).

In problem gambling treatment, cognitive-behavioral therapy has demonstrated success with significant reductions in gambling frequency and improved rates of abstinence among problem gamblers (Toneatto 2005; Toneatto and Millar 2004). Overall, the results of these cognitive treatment interventions have proved very promising, with findings that 80 % of problem gamblers successfully reduced their gambling to a non-problematic level after 12 months of treatment (Ferland et al. 2002; Ladouceur et al. 2000, 2001, 2003). Alternatively, efforts to prevent problem gambling behaviors by targeting cognitions have found limited success. In a review of current prevention programs, Williams et al. (2007) concluded that there are many prevention programs that provide marginal benefits, but there is no gold standard in gambling prevention. Despite mixed findings of young adults having an accurate understanding of the probabilities and odds (Delfabbro et al. 2006; Jefferson and Nicki 2003; Joukhador et al. 2004), evidence does not suggest that statistical knowledge, or the awareness of true randomness, can protect people from developing faulty gambling cognitions (Benhsain et al. 2004). Despite the limited empirical support for awareness/information campaigns, they remain the most commonly implemented model (Williams et al. 2007). This trend suggests that prevention efforts may suffer from an overemphasis on the role of cognitions, while minimizing the influence of other factors.

## Limitations

Participants from the MLSYA were young adults recruited from Manitoba, Canada. The demographic characteristics of the sample were not representative of either the Manitoban population or the Canadian young adult population. This limits the ability of the findings to be broadly applied to other populations.

Other potential limitations in this study include the transient nature of problem gamblers and attrition. Problem gambling has been demonstrated to have a transient nature, with natural recovery often coinciding with maturation into adulthood (Slutske et al. 2003; Winters et al. 2005). With high rates of natural recovery and low rates of treatment seeking (Slutske 2006), it is relatively common for gamblers to oscillate between pathological and non-pathological gambling states (Winters et al. 2002). Young adulthood is a period of development where multiple risk-taking behaviors are common, yet most of this age group will mature out of this behavior with age (Jessor 1998). This pattern suggests that the elevated pathological gambling prevalence seen in young adults (Shaffer and Hall 2000; Shaffer et al. 1999; Volberg 1996) may be reflective of a transient state. With this transient nature in mind, it is possible that the drop in gambling severity seen in this study’s longitudinal analyzes could be partially due to participants oscillating between gambling risk states.

## Conclusions

Our study provides insight towards the development of increased problem gambling severity in light of related faulty gambling cognitions. The findings of the current study highlight the simultaneous development of problem gambling and faulty beliefs although, over time, problem gambling severity may lead to erroneous beliefs more so than erroneous beliefs leading to problem gambling. In light of these findings, targeting erroneous beliefs as a means of preventing future problem gambling behavior may have limited effectiveness. This notion has been previously supported by findings that directing prevention efforts at gambling beliefs has a transitory effect on behaviors with no change from baseline observed after a 30 day period (Wohl and Sztainert 2010).
